# The Anatomical Basis of Paradoxical Masseteric Bulging after Botulinum Neurotoxin Type A Injection

**DOI:** 10.3390/toxins9010014

**Published:** 2016-12-30

**Authors:** Hyung-Jin Lee, In-Won Kang, Kyle K. Seo, You-Jin Choi, Seong-Taek Kim, Kyung-Seok Hu, Hee-Jin Kim

**Affiliations:** 1 Division in Anatomy and Developmental Biology, Department of Oral Biology, Human Identification Research Center, BK21 PLUS Project, Yonsei University College of Dentistry, Seoul 03722, Korea; leehj221@yuhs.ac (H.-J.L.); inwon25@hotmail.com (I.-W.K.); cyj7797@yuhs.ac (Y.-J.C.); hks318@yuhs.ac (K.-S.H.); 2 Modelo Clinic, Seoul 06011, Korea; doctorseo@hotmail.com; 3 Department of Oral Medicine, TMJ and Orofacial Pain Clinic, Yonsei University College of Dentistry, Seoul 03722, Korea; k8756050@yuhs.ac; 4 Room 601, Department of Oral Biology, Yonsei University College of Dentistry, 50-1 Yonseiro, Seodaemun-gu, Seoul 03722, Korea

**Keywords:** superficial part of masseter muscle, paradoxical masseteric bulging, botulinum neurotoxin Type A injection, lower facial contour

## Abstract

The aim of this study was to determine the detailed anatomical structures of the superficial part of the masseter and to elucidate the boundaries and locations of the deep tendon structure within the superficial part of the masseter. Forty-four hemifaces from Korean and Thai embalmed cadavers were used in this study. The deep tendon structure was located deep in the lower third of the superficial part of the masseter. It was observed in all specimens and was designated as a deep inferior tendon (DIT). The relationship between the masseter and DIT could be classified into three types according to the coverage pattern: Type A, in which areas IV and V were covered by the DIT (27%, 12/44); Type B, in which areas V and VI were covered by the DIT (23%, 10/44); and Type C, in which areas IV, V, and VI were covered by the DIT (50%, 22/44). The superficial part of the masseter consists of not only the muscle belly but also the deep tendon structure. Based on the results obtained in this morphological study, we recommend performing layer-by-layer retrograde injections into the superficial and deep muscle bellies of the masseter.

## 1. Introduction

The masseter is the most powerful jaw-closing muscle of the masticatory muscle group and is more developed in Asians than in Caucasians. Some Asian women in particular are interested in masseter reduction for improving the lower facial contour [1,2,3,4], and botulinum neurotoxin Type A (BoNT-A) injection has been widely performed in recent years for treating masseteric hypertrophy [5,6,7]. BoNT-A injection treatment is known to be a noninvasive and safe alternative therapy to surgical treatment for masseteric hypertrophy [8]. Since BoNT-A treatment was first reported by Moore (1994), several side effects have been found in numerous anatomical and clinical studies, including swelling, bruising, muscle weakness, and unexpected changes in the facial expression muscles [9,10].

Various anatomical studies have attempted to determine the most effective BoNT-A injection points and obtain optimal results while minimizing complications. Several of these studies have investigated the intramuscular nerve distribution, the motor nerve entry point of the masseteric nerve into the masseter, and the relationship between the parotid gland and the marginal mandibular branch of the facial nerve [11,12,13,14]. However, the rare side effect of paradoxical masseteric bulging after BoNT-A treatment for masseteric hypertrophy has not been investigated in any recent anatomical or clinical studies.

**Figure 1:**
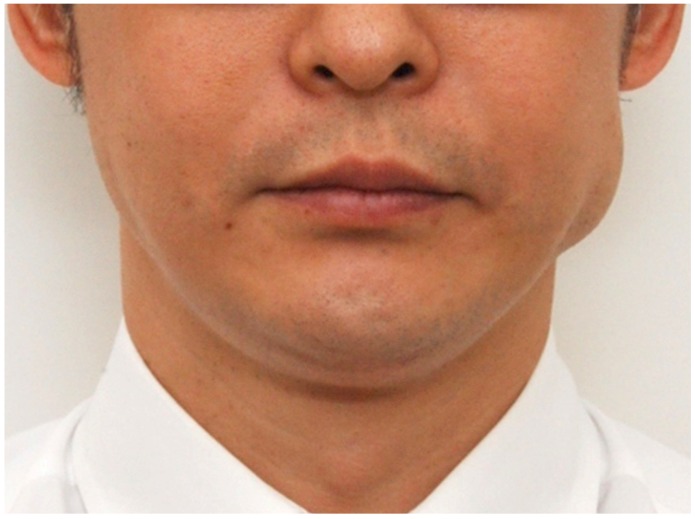
Photograph of paradoxical masseteric bulging. (Reproduced with permission from Seo K, Botulinum Toxin for Asians; Seoul Medical Publishing.)

Lee et al. (2012) reported the occurrence of paradoxical masseteric bulging after BoNT-A injection into a hypertrophied masseter (Figure 1) [15]. Even though this is a rare sequela, clinical trials focused on its anatomical clarification need to be performed. Therefore, the aim of this study was to determine the detailed anatomical structures of the superficial part of the masseter and to elucidate the boundaries and locations of the deep tendon structure within the superficial part of the masseter.

## 2. Results

**Figure 2:**
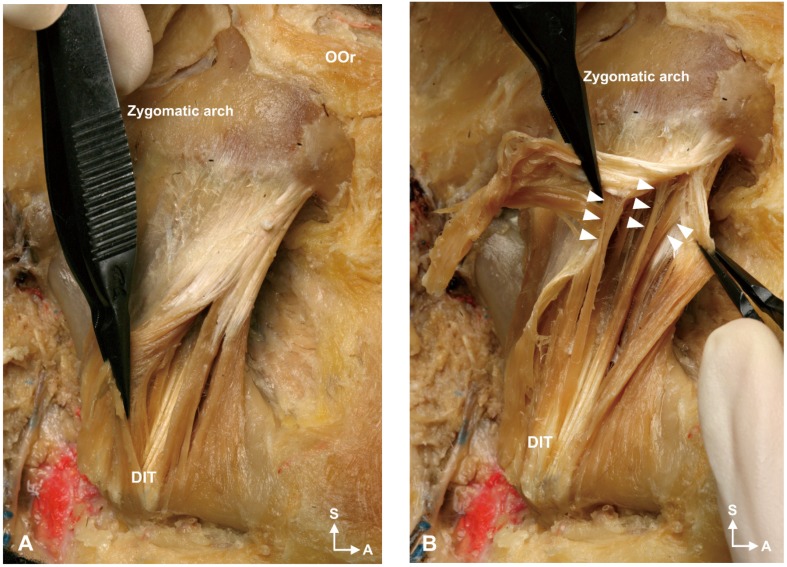
Detailed characteristics of the deep inferior tendon (DIT). (**A**) The DIT was located deep to the superficial muscle belly of the superficial part of the masseter. (**B**) The muscle fibers originated from the superficial aponeurosis of the masseter muscle, descended, and then changed into the tendon structure attaching to the inferior mandibular border. White arrowheads indicate the muscle fibers that originate from the deep to the superficial aponeurosis of the masseter muscle. A: anterior; S: superior; OOr: orbicularis oculi muscle.

The deep tendon structure was located deep in the lower third of the superficial part of the masseter. It was observed in all specimens and was designated as a deep inferior tendon (DIT). The muscle fibers originating from the superficial aponeurosis of the masseter descended and then changed into the DIT that attached to the inferior border of the mandible (Figure 2).

**Figure 3:**
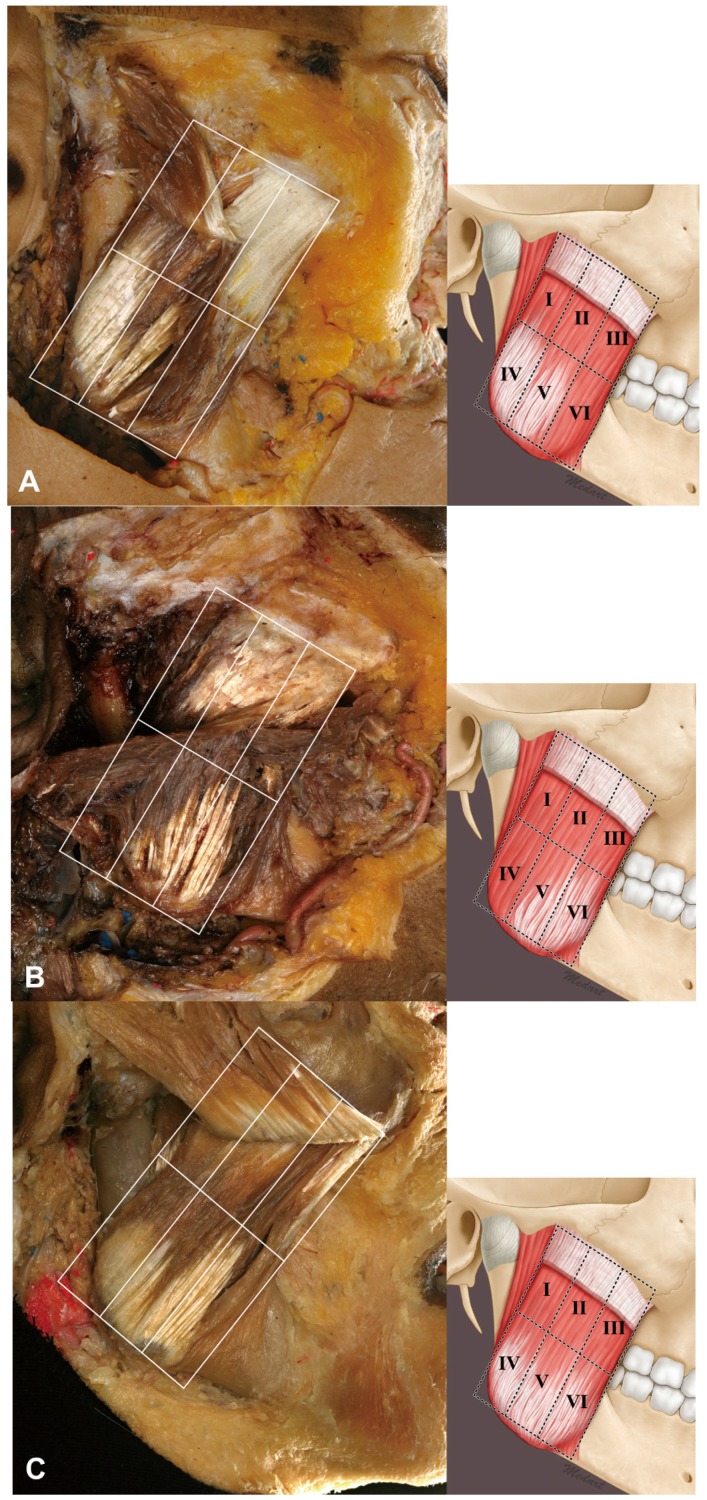
Classification of the DIT. The DIT can be found easily after removing the superficial muscle belly of the superficial part of the masseter. (**A**) Type A in which the DIT covers areas IV and V. (**B**) Type B in which the DIT covers areas V and VI. (**C**) Type C in which the DIT covers areas IV, V, and VI.

The relationship between the masseter and DIT could be classified into three types according to the coverage pattern: Type A, in which areas IV and V were covered by the DIT (27%, 12/44); Type B, in which areas V and VI were covered by the DIT (23%, 10/44); and Type C, in which areas IV, V, and VI were covered by the DIT (50%, 22/44) (Figure 3). Thus, the DIT typically covered the lower three compartments of the masseter. Type C (66.7%, 14/21) and Type A (39.1%, 9/23) were the most common types in both the Korean and Thai cadaveric specimens.

**Figure 4:**
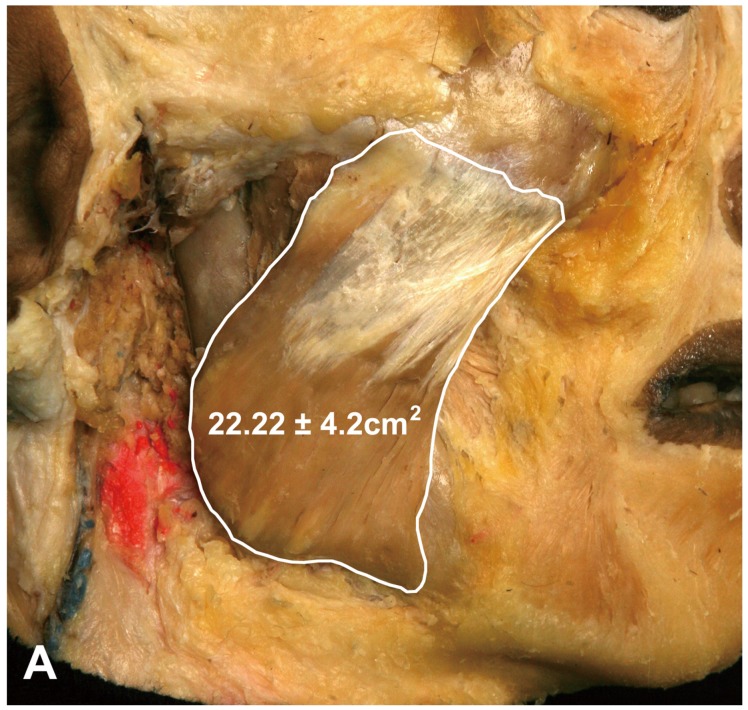

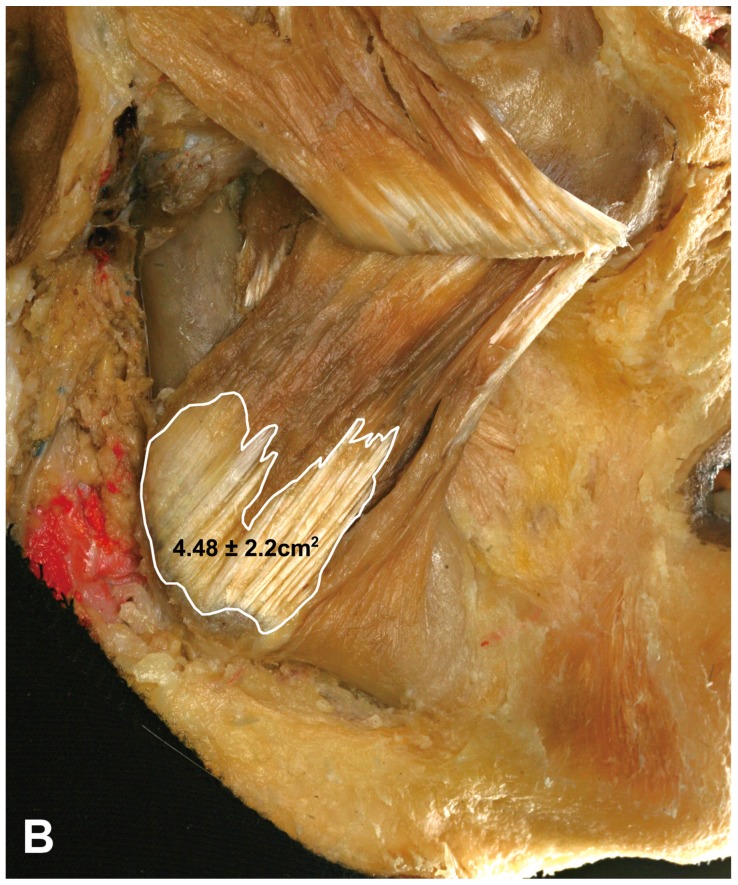
Proportions of the DIT in the superficial part of the masseter. The surface area of the superficial part of the masseter muscle was 22.22 ± 4.2 cm^2^ (**A**), and the DIT area within the masseter muscle was 4.48 ± 2.2 cm^2^ (**B**), hence constituting 22% of the superficial part of the masseter.

The proportion of the DIT in the superficial part of the masseter was measured using an image analysis program. The area of the superficial part of the masseter was 22.22 ± 4.2 cm^2^ (mean ± SD), and that of the DIT was 4.48 ± 2.2 cm^2^, thereby constituting approximately 22% of the superficial part of the masseter (19% in the Korean cadavers and 25% in the Thai cadavers) (Figure 4).

**Figure 5:**
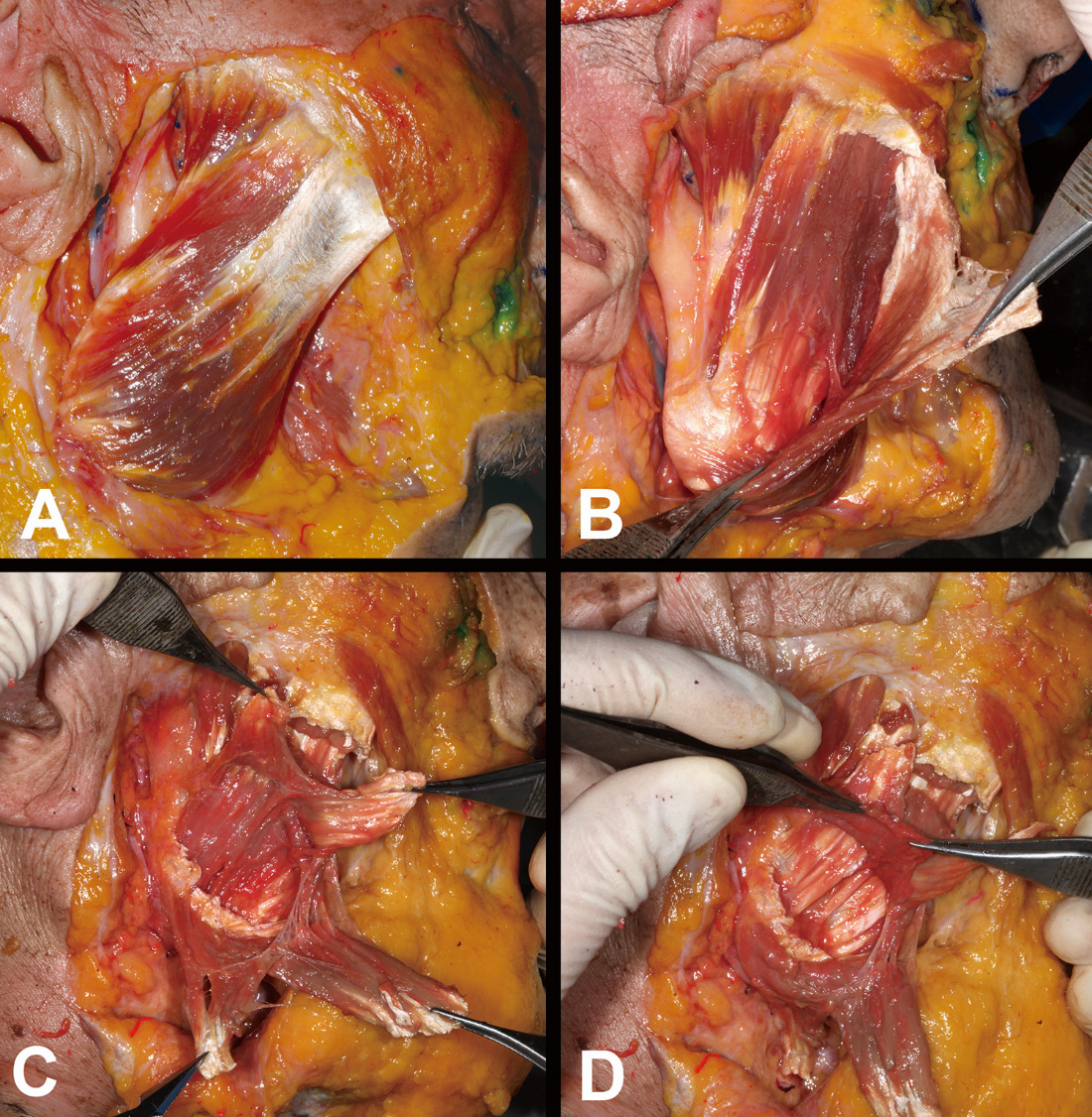
Serial dissections of the layered structures of the masseter muscle from superficial to deep. (**A**) Surfaces of the superficial part of the masseter. (**B**) The DIT was exposed after removing the superficial muscle belly and the aponeurosis of the superficial part of the masseter. (**C**) Another muscle belly was revealed after removing the DIT. (**D**) The middle and deep parts of the masseter muscle were attached at the lateral surface of the mandible and the periosteum.

The DIT arose deep in the superficial aponeurosis of the masseter, ran downward, and then changed into a tendon attaching to the inferior border of the mandible. Another muscle fiber that originated from the zygomatic arch was present deep in the DIT. The middle and deep muscle fibers of the masseter were observed to be deeper than this muscle fiber. In the deeper part, the periosteum was found attached to the lateral aspect of the mandible (Figure 5).

## 3. Discussion

**Figure 6:**
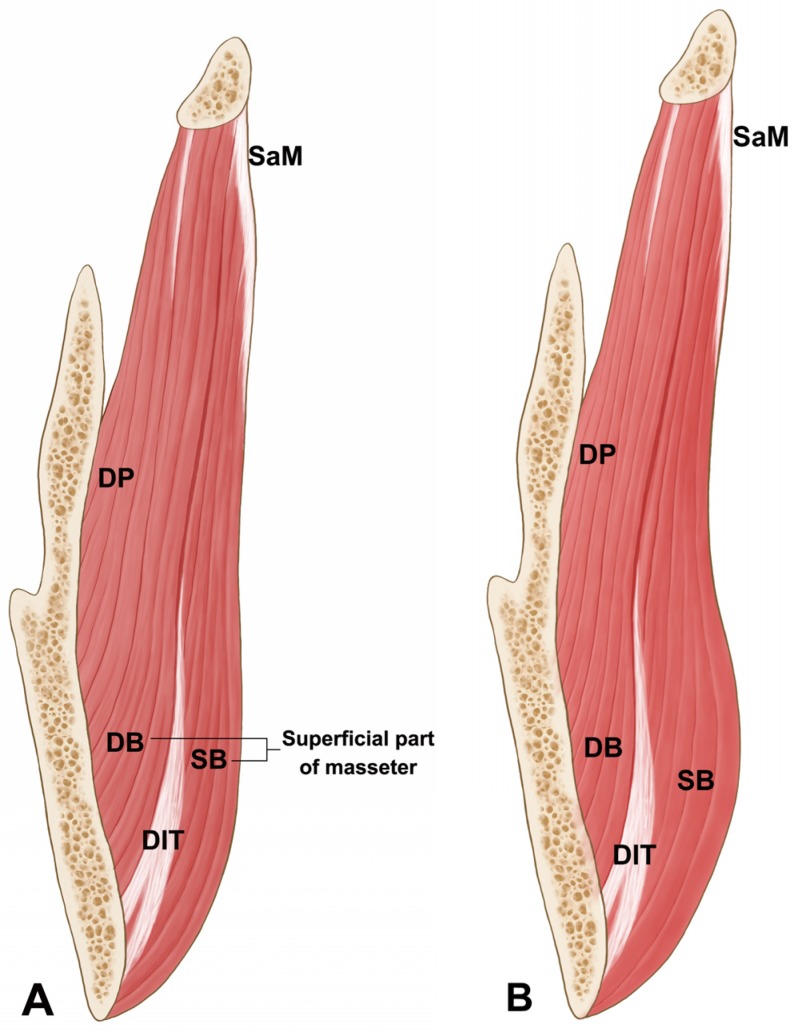
The illustration of the coronal section of the masseter from superficial to deep (**A**) and the expected muscle feature showing the masseteric bulging after the BTX-A injection into the masseter muscle during clenching (**B**). When masseter contracts, paradoxical masseteric bulging may occur at the superficial belly of the superficial part of the masseter because the DIT blocks the toxin’s diffusion. (SB: superficial muscle belly of superficial part of the masseter; DB: deep muscle belly of superficial part of the masseter; DP: deep part of the masseter; DIT: deep inferior tendon; SaM: superficial aponeurosis of the masseter).

This is the first anatomical study to propose a mechanism underlying paradoxical masseteric bulging after BoNT-A injection. Based on the observations made in this study, it was hypothesized that a broad tendon structure (i.e., the DIT) located within the superficial layer of the masseter may prevent the spreading of BoNT-A into the entire layer of the superficial muscle belly (Figure 6A). This would result in the deeper muscle belly of the superficial masseter usually being paralyzed, and in rare cases bulging of a part of the superficial muscle belly that is unaffected by BoNT-A due to the presence of the DIT (Figure 6B). The DIT originated deep to the aponeurosis of the superficial part of the masseter in every case.

The masseter has been previously described as consisting of three layers: superficial, middle, and deep (zygomaticomandibularis muscle) [12]. The superficial layer has usually been considered to comprise a muscle belly only; however, we found that the superficial layer has quite a complicated structure composing of a deep and broad tendon within the muscle belly in the present study. The organization of the entire masseter was reported in 2000, and those results were generally comparable to those obtained in the present study [16].

The DIT was located and formed deep to the superficial part of the masseter and broadly attached to the lower third of the mandibular ramus and the inferior border of the mandible (Figure 4). Moreover, an additional thin muscle belly was observed deep to the DIT, and the insertion of the deep part of the masseter and periosteum was observed underneath this deeper thin muscle layer. Based on our findings, the superficial part of the masseter appears as lamellar structures consisting of the overlapped muscle belly and deep tendon structure. Thus, the superficial layer to the deep layer of the masseter were organized in the following order: superficial muscle belly of the masseter, DIT, deeper muscle belly, and insertion of the middle and deep parts of the masseter and periosteum (Figure 6).

The most common injection-based method for treating masseteric hypertrophy involves injecting BoNT-A either deep into the lower third of the masseter or where it bulges the most [5,17,18]. This method is designed to avoid damage to the facial nerve and the superficial facial muscles such as the risorius and zygomaticus minor muscles, which could result in unexpected changes in facial expression and paralysis of the buccinator and pterygoid muscles [2,19,20,21].

The DIT was located in the lower third of the masseter in every case, but its morphology varied slightly between the Korean and Thai specimens. The most common type in the Korean cadavers was Type C, which covered areas IV, V, and VI (66.7% of specimens), while Type A, which covered areas IV and V (39.1% of specimens), was the most common in the Thai cadavers. These observations imply that the injection techniques applied to Korean and Thai patients need to be modified in order to optimize the treatment of masseteric hypertrophy. Even though Type C (areas IV, V, and VI) is the most common type in Koreans, area VI is known to be a vulnerable region since it is covered by the risorius muscle.

Paradoxical masseteric bulging occurs in rare cases after BoNT-A injections into the masseter [15] and has been discussed only anecdotally in the clinical field, with no detailed anatomical information available about the fundamental underlying cause. The current results show clearly that the DIT can prevent the toxin from spreading into the entire superficial masseter during the BoNT-A injection procedure. That is, the DIT acts as a window within the superficial masseter that can confine the injected toxin to within the deeper muscle belly.

Ultimately, the superficial part of the masseter consists of not only the muscle belly but also the deep tendon structure. Considering that the deep tendon structure was present in every specimen of the present study, physicians should be aware of this tendon prior to treating masseteric hypertrophy and improving lower face contouring using BoNT-A injections. Knowledge of this critical anatomical information will help to prevent paradoxical masseteric bulging. Based on the results obtained in this morphological study, we recommend performing layer-by-layer retrograde injections into the superficial and deep muscle bellies of the masseter (Figure 6).

## 4. Materials and Methods

Signed and informed consent was obtained from the volunteer. Forty-four hemifaces from Korean (21 hemifaces, 10 left and 11 right; mean age 79.9 years) and Thai (23 hemifaces, 11 left and 12 right; mean age 68.6 years) embalmed cadavers were dissected to analyze the morphological patterns, with 30 of them used to measure the detailed location of the deep tendon structure of the masseter.

The skin of the midface was carefully removed, and the superficial musculoaponeurotic system (SMAS), parotid gland, and masseteric fascia were dissected to reveal the entire masseter. The superficial part of the masseter was dissected to expose and observe the morphological patterns and location of the deep tendon structure.

The surface of the superficial part of the masseter was classified into six equivalently sized rectangular areas (designated I to VI) to enable the portion covered by the deep tendon structure to be delineated. Areas I–III and IV–VI were designated as the upper and lower three compartments, respectively. The location of the deep tendon structure was categorized according to its position with respect to these six compartments.

The proportion of the deep tendon structure in the superficial part of the masseter was measured using an image analysis program (I-solution, IMTechnology, Coquitlam, BC, Canada).
